# Explaining increased locoregional failure following total neoadjuvant treatment in RAPIDO

**DOI:** 10.1093/bjs/znag095

**Published:** 2026-07-15

**Authors:** Per J Nilsson, Ilaria Prata, Max D Tanaka, Alice M Couwenberg, Boudewijn van Etten, Geke A P Hospers, Bengt Glimelius, Cornelius J H van de Velde

**Affiliations:** Department of Pelvic Cancer, Karolinska University Hospital, Stockholm, Sweden; Department of Surgery, Leiden University Medical Centre, Leiden, The Netherlands; Department of Radiation Oncology, Amsterdam University Medical Centre, Amsterdam, The Netherlands; Department of Radiation Oncology, Netherlands Cancer Institute, Amsterdam, The Netherlands; Department of Surgery, University of Groningen & University Medical Centre Groningen, Groningen, The Netherlands; Department of Medical Oncology, University of Groningen & University Medical Centre Groningen, Groningen, The Netherlands; Department of Immunology, Genetics and Pathology, Uppsala University, Uppsala, Sweden; Department of Surgery, Leiden University Medical Centre, Leiden, The Netherlands

The RAPIDO trial, currently the largest trial on total neoadjuvant treatment (TNT) in rectal cancer, met its primary end-point (disease-related treatment failure), reported fewer distant metastases, and doubled the rate of pathological complete response. However, an increased rate of locoregional recurrence (LRR) after long-term follow-up has caused concern, debate and, among some, reluctance to include short-course radiotherapy (SCRT) in a TNT schedule.

Recently, two publications in the *BJS* provide the foundation for a plausible explanation of the increased LRR rate^1,2^. A post-hoc analysis of RAPIDO data showed that the difference in LRR between TNT and chemoradiotherapy (CRT) was statistically significant only after sphincter-preserving surgery and not abdominoperineal excision (APE)^1^. Furthermore, the difference was evident in Dutch patients but not among Swedish counterparts. Both these observations raise questions as to how neoadjuvant therapy can exert a variable effect depending on where and what type of surgery was performed. Notable differences between the countries were that APE was used more often in Sweden, and the distal resection margin (DRM) was ‘involved’ (≤10 mm) after sphincter-preserving surgery more often in Dutch patients. Among patients with a DRM exceeding 10 mm no difference in LRR was reported, whereas among patients with a narrower DRM the difference was obvious (CRT: 1.8% *versus* TNT: 25.4%; hazard ratio 15.51 [2.02–119.35]).

The essentially nationwide Swedish non-randomized study LARCT-US used an abbreviated RAPIDO schedule (SCRT and 4 CAPOX cycles) in truly locally advanced rectal cancer (cT4: 56%, MRF+: 77%, lateral lymph nodes: 23%), and 5-year results showed an LRR rate of only 6% in the cohort of 437 patients^2^. Indeed, an increased LRR rate is not a universal finding in TNT trials using SCRT, and the STELLAR trial reported similar LRR rates between study arms^3^. Conversely, a 3-year local relapse rate of 16% after surgery (probably 10–12% counting all treated) has been reported after TNT using induction chemotherapy and CRT (cT4: 18%)^4^.

Irrespective of whether CRT or SCRT was used in a TNT schedule, tumour downsizing/staging is consistently reported more pronounced following TNT compared to CRT. However, downsizing can include tumour fragmentation and minute clusters of viable cancer cells, undetectable on restaging MRI, may persist in the proximity of the visible remnant tumour^5^. Thus, it can be hypothesized that a narrower DRM carries a risk of leaving viable tumour fragments behind and this may, in turn, lead to LRR (*Fig. 1*). Commonly, the final decision on surgical procedure is based on the restaging MRI and an apparently good, albeit not complete, response may lead surgeons to opt for sphincter-preserving surgery. Because the remnant tumour clusters are undetectable this may result in an insufficient DRM. It is also conceivable that the wish to avoid a permanent stoma may differ in different cultural contexts.

**Figure 1:**
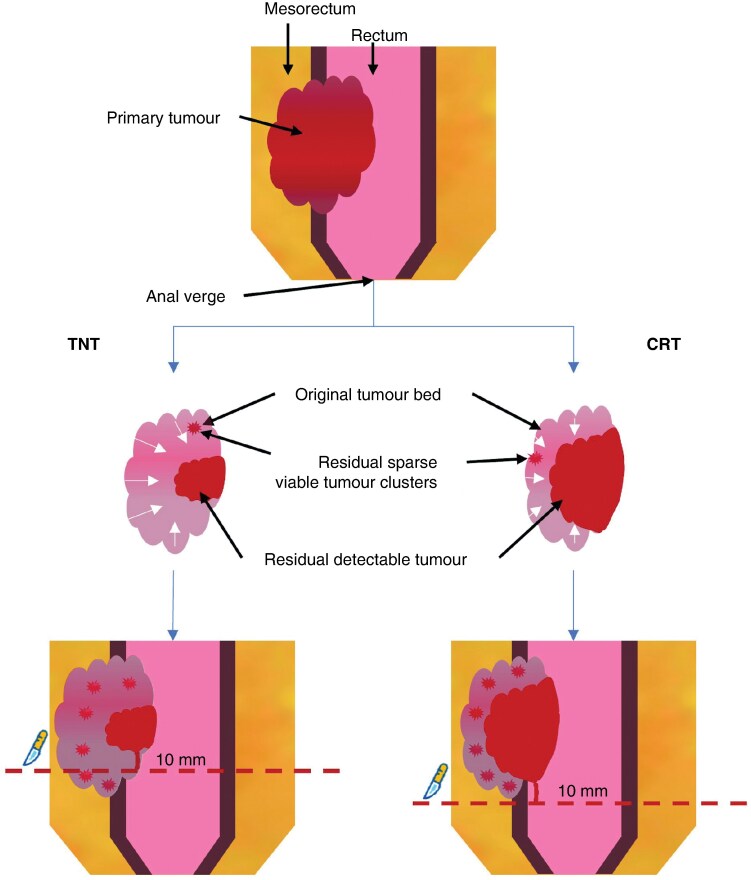
A distal margin of ≤10 mm below the remaining detectable tumour may be insufficient to achieve low LRR rates after TNT

Secondary intervention bias or co-intervention bias can occur in trials where additional treatment decisions and interventions are performed after the initial randomization^6^. In the RAPIDO trial, the surgical decision-making was after restaging MRI in both treatment arms. Following TNT, a decision to perform APE was more common in Sweden compared to the Netherlands (46% *versus* 30%, *P* = 0.003) despite similar patients being randomized, resulting in significantly fewer patients with a narrow DRM (8% *versus* 29%)^1^.

In conclusion, TNT including SCRT for locally advanced rectal cancer can result in excellent locoregional control as reported from LARCT-US^2^. The increased rate of involved DRM reported in Dutch RAPIDO patients may be the result of co-intervention bias (based on a well-meant ambition to avoid APE), and the rate of LRR in these patients therefore represents a surgical, and not an oncological, problem.

## Data Availability

Data related to this research letter are based on previously published work.
